# Shame on Me! Self-Conscious Emotions and Big Five Personality Traits and Their Relations to Anxiety Disorders Symptoms in Young, Non-Clinical Adolescents

**DOI:** 10.1007/s10578-017-0747-7

**Published:** 2017-07-14

**Authors:** Peter Muris, Cor Meesters, Mike van Asseldonk

**Affiliations:** 1 Maastricht University & Virenze-RIAGG, Maastricht, The Netherlands; 2 Stellenbosch University, Stellenbosch, South Africa; 3 Department of Clinical Psychological Science, Maastricht University, P.O. Box 616, 6200 MD Maastricht, The Netherlands

**Keywords:** Shame and guilt, Self-conscious emotions, Big Five personality traits, Anxiety disorders symptoms, Adolescents

## Abstract

This study explored the relations between self-conscious emotions, personality traits, and anxiety disorders symptoms in non-clinical youths. One-hundred-and-eighteen adolescents aged 12–15 years completed the brief shame and guilt questionnaire for children (BSGQ-C) and items of the youth self-report (YSR) to measure shame and guilt, the big five personality questionnaire for children, and the youth anxiety measure for DSM-5. Results for shame indicated that this self-conscious emotion—either measured by the BSGQ-C or the YSR—was uniquely and positively associated with a broad range of anxiety disorders symptoms, and correlated positively with neuroticism and negatively with extraversion. Guilt did not show significant associations with anxiety disorders symptoms once controlling for the influence of shame, and links with personality traits varied dependent on the assessment instrument that was used (BSGQ-C or YSR). Finally, when controlling for neuroticism and extraversion, shame consistently remained a significant correlate of anxiety disorders symptoms. Altogether, these results add to the growing body of evidence indicating that high levels of shame are clearly associated with anxiety pathology.

## Introduction

The self-conscious emotions of shame and guilt are experienced when a person violates some moral or social standard while realizing that this transgression is noticed by other people. The feelings of tension, remorse, and regret associated with these emotions prompt the individual to correct and compensate for the inappropriate behavior, restoring the relationship with those who were disturbed or offended by the reprehensible action [1]. Shame and guilt have clear similarities: they are both negatively valenced emotions that serve the adaptive purpose of promoting people’s compliance to moral and social rules, thereby streamlining social interactions and intimate relationships [2]. However, a closer inspection also reveals that these emotions are quite different in nature. That is, whereas guilt is concerned with the negative evaluation of a specific behavior (“I did *that* wrong”) resulting in a desire to confess, apologize, and repair, shame pertains to the negative evaluation of the self (“*I* did that wrong”) causing a desire to vanish, escape, or strike back [3]. Tangney [4] was among the first scholars to point out that this distinction between guilt and shame may also be relevant for our understanding of psychopathology. She argued that guilt should be primarily viewed as a “good” emotion; because of its positive associations with morality and empathy, this self-conscious emotion probably prevents the development of externalizing (i.e., disruptive behavior) problems. In contrast, Tangney labeled shame as “bad and ugly”, as excessively high levels of this self-conscious emotion likely promote inferiority, self-punishment, and defensive aggression, and in its wake would make individuals more susceptible for developing both internalizing (i.e., emotional) and externalizing problems.

More than a quarter of a decade later, evidence on the relations between the self-conscious emotions of shame and guilt and psychopathology has steadily accumulated. Most studies have been conducted in the areas of depression [5], which belongs to the category of internalizing problems, and anger and aggression [6], which are characteristic features of externalizing problems. The research generally demonstrates that shame is indeed positively linked to depression as well as anger/aggression. The results obtained for guilt are more complicated. A meta-analysis by Kim et al. [5] showed that this self-conscious emotion is also positively related to depression and in the Diagnostic and Statistical Manual of Mental Disorders [7] “excessive or inappropriate guilt” is even one of the defining features of this disorder, but there is also evidence indicating that once controlling for the overlap with shame, guilt is no longer significantly related to depression [2]. Meanwhile, research has confirmed that guilt displays the predicted negative association with anger/aggression [2, 8]. Altogether, findings are nicely in keeping with Tangney’s [4] notion that high shame levels are associated with an increased proneness to a broad range of psychopathological symptoms, whereas ‘pure’ guilt (i.e., guilt that is not fused with shame) essentially is a benign emotion that is unrelated to psychopathology. Only in the case that guilt levels are extremely low, a heightened susceptibility to develop externalizing problems is found.

In recent years, researchers have begun to explore the association between shame/guilt and anxiety disorders symptoms. Based on Tangney’s [4] original ideas, it can be hypothesized that again the emotion of shame is especially relevant for this type of psychopathology. In view of the fact that shame primarily occurs in a social context and is associated with a heightened sense of self-awareness in combination with an increased likelihood of being negatively evaluated by others, it is first of all plausible to assume that this self-conscious emotion is strongly linked to symptoms of social anxiety disorder. Indeed, the available evidence is indicating that shame (but not guilt) is positively associated with symptoms of social anxiety, and that this appears true in both clinical and non-clinical adult populations [9–15]. Further, there is also research showing that shame is positively related to other types of anxiety symptoms. For instance, several studies, again primarily conducted with adult samples, found evidence to suggest that shame (and again not guilt) plays a role in worry and generalized anxiety disorder [16–18], and there is one investigation [19] that has reported a positive link between shame and dispositional anxiety as assessed by the Spielberger State-Trait Anxiety Inventory [20]. The relation between shame and other (non-social) anxiety symptoms is on first sight more difficult to explain, although it can be noted that this self-conscious emotion is associated with a sense of inadequacy and weakness that might undermine one’s self-efficacy when confronted with stressful and threatening situations thereby paving the way for feelings of fear and anxiety [21]. Alternatively, at a behavioral level, shame is associated with a tendency to vanish and escape, which of course shows strong resemblance with avoidance, one of the key mechanisms operating in the maintenance of fear and anxiety pathology [22].

Most anxiety disorders have an early age-of-onset [23], and therefore it makes sense to study the etiology of this type of psychopathology in the childhood and teenage years [24]. In the case of a presumed role of self-conscious emotions, the developmental stage of adolescence is particularly relevant because teenagers on the one hand have a more advanced cognitive capacity allowing for self-reflection [25] and social perspective taking [26], while on the other hand strive to develop a valued sense of the self and one’s identity irrespective of biological, social, and emotional challenges [for a discussion of the normative development of self-conscious emotions, see [2, 8]). This will make them more prone to experience self-conscious emotions in general [27], and shame in particular [28]. Despite the plausible link between self-conscious emotions and anxiety in young people, only a handful of studies have been conducted. A first study by Muris et al [29] examined the relations between shame/guilt, behavioral inhibition (as an index of anxiety proneness [30]), and anxiety disorders symptoms in non-clinical youths aged 8–13 years (of which some were in the developmental stage of (pre-) adolescence). Results indicated that shame (but not guilt) was uniquely related to behavioral inhibition and a broad range of anxiety disorder symptoms. Moreover, shame remained a significant predictor of total anxiety symptoms and symptoms of generalized anxiety disorder, after controlling for behavioral inhibition. In a second investigation, Paulus et al. [31] tested the unique roles of shame (guilt was not included in this study) and two other vulnerability factors, namely psychological inflexibility and emotion dysregulation, as mediators in the relation between neuroticism and anxiety symptoms in a sample of 97 inpatient adolescents aged 12–17 years. The results showed that shame was the only significant mediator, signifying an indirect path between neuroticism and anxiety. A final study by Muris et al. [32] assessed levels of self-conscious emotions as assessed via three informants (i.e., children themselves, parents and teachers) in 1000 clinically referred youths with anxiety disorders and other types of internalizing (depression), externalizing (disruptive behavior disorders) and developmental (autism) psychopathology (of whom 383 were aged between 12 and 18 years). The results revealed that both shame and guilt were elevated in children and adolescents with internalizing problems, but that this was most consistently (i.e., across all informants) true for the young patients with anxiety disorders as their primary diagnosis. All these findings underline the relevance of self-conscious emotions, especially shame, in anxiety pathology in youths. In line with previous work in adults, guilt was found to be largely unrelated to anxiety, and in case such a link did emerge [32], this was probably due to shared variance with shame [2, 8].

Personality traits are also strong correlates of anxiety symptoms and disorders. For example, Kotov et al [33] conducted a meta-analysis on the associations between the Big Five (i.e., neuroticism, extraversion, agreeableness, conscientiousness, and openness/intellect) and Big Three (i.e., neuroticism, extraversion, psychoticism) personality factors and a broad range of anxiety and phobic disorders in adults, and found that most anxiety problems are characterized by high levels of neuroticism and low levels of extraversion. Similar patterns have been noted in research with children and adolescents [34], and there is also evidence that the aforementioned anxiety-prone temperament style of behavioral inhibition [30] is a mix of the personality traits neuroticism and (low) extraversion [35, 36]. Interestingly, personality traits also show meaningful links with self-conscious emotions. That is, because shame is often associated with negative feelings about the self and submissive behavior in social situations, it is not surprising that studies found that this self-conscious emotion is positively related to neuroticism but inversely associated with extraversion [14, 37–39]. In contrast, guilt being associated with empathy and prosocial behavior is shown to be linked with the more positive personality trait of agreeableness [37, 38, 40]. Note however that most studies on the links between self-conscious emotions and personality traits have been conducted in adults.

The present study further explored the relations between self-conscious emotions, Big Five personality traits, and anxiety symptoms in youths. The investigation builds on previous work and especially on the study of Paulus et al. [31], but at the same time is also novel as it deviates from this research in three ways: (1) Whereas Paulus et al. relied on a sample of adolescent inpatients, the present study was conducted in typically developing adolescents thereby providing the opportunity to establish whether findings are comparable for clinical and non-clinical youths; (2) While Paulus et al. exclusively focused on shame, the present study also included guilt, which enabled us to further explore the unique features of both self-conscious emotions in young people and to investigate their relative importance in anxiety symptoms; and (3) Paulus et al. only examined the personality trait of neuroticism. The present study investigated all Big Five personality traits in relation to self-conscious emotion and anxiety symptoms, which is relevant as earlier research has shown that extraversion is at least as important as neuroticism within the context of self-conscious emotions and anxiety.

Thus, the current investigation was conducted in 118 non-clinical adolescents who completed a set of questionnaires for measuring all relevant constructs. It was hypothesized that: (a) proneness to shame, but not guilt, would be positively associated with symptom levels of various anxiety disorders (i.e., major anxiety disorders among which social anxiety disorder and generalized anxiety disorder, and phobic disorders such as specific phobias), but given the interpersonal function of self-conscious emotions a particularly strong link was expected to be found with symptoms of social anxiety; (b) shame would be positively related to the personality trait of neuroticism and negatively to the trait of extraversion, whereas guilt was anticipated to be positively related to the trait of agreeableness; and (c) shame would make an independent contribution to various types of anxiety symptoms beyond the influence of the personality traits of neuroticism and extraversion.

Besides these main research topics, a number of additional issues were addressed in this study. To begin with, we explored gender differences in self-conscious emotion, with the expectation that (d) girls would display somewhat higher levels of shame and guilt than boys [41]. Note that this is particularly relevant within the context of anxiety problems, for which a skewed female to male ratio has been reported [7]. A further aim was to examine the psychometric properties of the two assessment instruments that were used to measure shame and guilt in children and adolescents, namely the brief shame and guilt questionnaire for children (BSGQ-C) [42] and a number of items taken from the youth self-report (YSR) [43]. Until now, the evidence on the validity of these scales for measuring self-conscious emotions is quite limited [32, 42], and so the present study provided the opportunity to demonstrate that (e) the BSGQ-C and the YSR items have sufficient convergent validity and correlate in a similar, theoretically meaningful way with the personality traits of neuroticism and extraversion and anxiety disorders symptoms.

## Method

### Participants and Procedure

Participants for this study were recruited at the Udens College, a regular secondary school in Uden, The Netherlands. Two-hundred-and-twenty students on this school were approached to take part in this study by sending them and their parents an information letter along with an informed consent form. Eventually, 118 of the adolescents and their parents (53.6%) responded positively to this invitation, and subsequently the young participants completed the set of questionnaires (see below) during regular classes. The final sample consisted of 53 boys and 65 girls, who had a mean age of 13.31 years (*SD* = 0.67, range 12–15 years) and primarily were from original Dutch descent (>90%). The non-Dutch youth came from families with an Indonesian, Antillean, Surinam, Moroccan, or Turkish background. Due to school constraints, no other information regarding the educational level and socioeconomic status of the participants was available.

### Assessment

The BSGQ-C has been developed by Novin and Rieffe [42] and consists of 12 brief vignettes of which six scenarios measure shame (e.g., “You fall with your bike on the pavement. People stop to watch”) and six scenarios assess guilt (e.g., “You want to go home quickly. You see the little girl from next door dropping all her marbles. You don’t help her because you are in a hurry”). Following each scenario, children are asked to rate how much shame or guilt they would feel using a 3-point scale (1 = Not at all, 2 = A little, 3 = A lot). Psychometric evaluation of the BSGQ-C [42] in a sample of 219 children and young adolescents aged 8–14 years has indicated that the measure is reliable in terms of internal consistency, with Cronbach’s alphas of 0.80 and 0.76 for the shame and guilt subscales respectively. Further, a factor analysis produced a clear two-factor solution [42], with all shame items loading on one factor and all guilt items loading on the other factor, which supports the construct validity of the measure. Finally, evidence for the concurrent validity of the BSGQ-C was obtained through theoretically meaningful correlations with scales measuring internalizing and externalizing symptoms. That is, the BSGQ-C shame subscale correlated positively with symptoms of social anxiety and worry, whereas the guilt subscale correlated negatively with conduct problems and aggression [42].

Three items taken from the *youth self-report* (YSR) version of the Achenbach System of Empirically-Based Assessment (ASEBA) [43, 44] were used as an alternative index of youth’s self-conscious emotions. These were item 26 “Lacks guilt”, item 52 “Feels very guilty”, and item 71 “Self-conscious, easily ashamed” representing respectively the concepts of lack of guilt, guilt, and shame [32].

The *Big five questionnaire for children* (BFQ-C) [45] is a 65-item questionnaire for measuring the five basic factors of personality (i.e., the Big Five) in children and adolescents: (1) extraversion which has to do with outgoing, talkative, energetic behavior (e.g., “I like to meet with other people”), (2) agreeableness which reflects concern and sensitivity towards others and their needs (e.g., “I share my things with other people”), (3) conscientiousness which has to do with dependability, orderliness, precision, and the fulfilling of commitments (e.g., “I do my job with care and attention”), (4) neuroticism which pertains to a proneness to experience negative feelings (e.g., “I get nervous for silly things”), and (5) intellect/openness which is concerned with intellectual functioning, creativity, and a broad social and cultural interest (e.g., “I know many things”). Items have to be scored on a five-point Likert scale ranging from 1 = *almost never* to 5 = *almost always*. Individual item scores are combined to yield a total score for each of the five factors. Clear support has been found for the psychometric qualities of the BFQ-C in youths from various countries [45–50].

The *youth anxiety measure for DSM-5* (YAM-5) [51] assesses anxiety disorders symptoms of children and adolescents in terms of the latest version of this psychiatric classification system. The scale consists of 50 items that are divided into two parts. The first part of the YAM-5 (or YAM-5-I) contains 28 items assessing symptoms of the major anxiety disorders, including separation anxiety disorder (e.g., “I get frightened if my parents leave the house without me”), selective mutism (e.g., “At school I don’t speak to the teacher at all”), social anxiety disorder (e.g., “I find it scary to eat or drink if other people are looking at me”), panic disorder (e.g., “I suffer from anxiety or panic attacks”), and generalized anxiety disorder (e.g., “I worry about a lot of things”). The second part (YAM-5-II) contains 22 items referring to symptoms of phobias: animal phobias (e.g., “I’m afraid of snakes”), natural environment phobias (e.g., “I am afraid of heights”), blood-injection-injury phobias (e.g., “I am afraid of getting an injection”), situational phobias/agoraphobia (e.g., “I am afraid when travelling by bus or train”), and other phobias (e.g.,“I am afraid of people who are dressed up in costumes”). The self-report version asks children to respond to each item using a four-point Likert-type scale with 0 = never, 1 = sometimes, 2 = most of the time, and 3 = always. Ratings are summed to yield total and subscale scores, with higher scores reflecting higher levels of anxiety disorder and phobia symptoms. So far, research has demonstrated that the YAM-5 is a reliable scale, with satisfactory parent–child agreement and good concurrent validity as evinced by positive correlations with other measures of fear, anxiety, and depression, and clinical diagnosis of an anxiety disorder [51–53].

### Statistical Analysis

The Statistical Package for Social Sciences (SPSS, Version 21) was used to compute descriptive statistics, to conduct reliability analyses of various questionnaires, and to examine associations among study variables by means of correlations. In this article, we report partial correlations that corrected for the influence of gender and age (which appeared to have influence on some variables; see below). However, it is important to note that the uncorrected correlations were highly similar and that comparable results would have been obtained when not controlling for these demographic variables. Steiger’s [54] method was used for comparing the strength among correlation coefficients. Hierarchical regression analyses were conducted to examine the unique contributions of shame and guilt to anxiety disorders symptoms beyond the effects of Big Five personality traits.

## Results

### Reliability and Age/Gender Effects

Table 1 displays some descriptive statistics of various scales that were administered in the sample of adolescents. Three conclusions can be drawn from this table. First, a number of significant gender differences were found. As expected, girls scored higher on both the shame and the guilt subscales of the BSGQ-C [*t*(116)’s being 2.10 and 2.71, respectively, both *p*’s < 0.05] as compared to boys. In addition, girls also displayed higher scores on BFQ-C agreeableness [*t*(116) = 3.35, *p* < .01] and YAM-5 anxiety symptoms [all *t*(116)’s ≥ 2.30, *p*’s < 0.05], but lower scores on BFQ-C extraversion [*t*(116) = 3.10, *p* < .01] than did boys. Second, for some variables, statistically significant negative associations were documented with age. As youths were older, they reported lower levels of shame on the BSGQ-C, neuroticism, and anxiety disorder and phobia symptoms. Third, questionnaires were in general reliable in terms of internal consistency, with Cronbach’s alphas being 0.72 and 0.70 for BSGQ-C shame and guilt, respectively, and ranging between 0.74 and 0.84 for BFQ-C and between 0.80 and 0.90 for YAM-5 scales. Note that for the YSR no reliability coefficients could be computed because these indices of self-conscious emotions only consist of a single item.

**Table 1:** Descriptive statistics (means, standard deviations, gender differences, relation with age, and internal consistency coefficients) of various questionnaires included in the present study

	Total sample (*N* = 118)	Boys (*n* = 53)	Girls (*n* = 65)	*r* with age	Cronbach’s α
BSGQ-C Shame	12.29 (2.61)	11.74 (2.45)_a_	12.74 (2.67)_b_	− 0.23*	0.72
BSGQ-C Guilt	13.42 (2.24)	12.81 (2.08)_a_	13.91 (2.27)_b_	−0.07	0.70
YSR Shame	0.73 (0.68)	0.64 (0.68)_a_	0.80 (0.67)_a_	−0.14	–^‡^
YSR Guilt	0.77 (0.61)	0.70 (0.54)_a_	0.83 (0.65)_a_	−0.10	–^‡^
YSR Lack of guilt	0.97 (0.60)	0.89 (0.54)_a_	1.03 (0.64)_a_	0.15	–^‡^
BFQ-C Extraversion	36.45 (5.46)	38.11 (4.56)_a_	35.09 (5.78)_b_	−0.02	0.78
BFQ-C Agreeableness	38.15 (5.30)	36.42 (4.59)_a_	39.57 (5.45)_b_	0.01	0.84
BFQ-C Conscientiousness	32.25 (5.01)	31.45 (4.62)_a_	32.91 (5.25)_a_	0.05	0.74
BFQ-C Neuroticism	22.50 (4.94)	22.08 (4.96)_a_	22.85 (4.94)_a_	−0.19†	0.75
BFQ-C Openness/intellect	31.20 (5.52)	31.25 (4.66)_a_	31.17 (6.16)_a_	− 0.05	0.75
YAM-5 Total anxiety	23.25 (12.75)	18.58 (10.46)_a_	27.05 (13.25)_b_	−0.32**	0.90
YAM-5-I Anxiety disorders	13.55 (7.96)	11.72 (6.19)_a_	15.05 (8.93)_b_	−0.31*	0.88
YAM-5-II Phobias	9.69 (6.62)	6.87 (5.55)_a_	12.00 (6.56)_b_	−0.24*	0.80

*BSGQ-C* Brief shame and guilt questionnaire for children, *YSR* youth self-report, *BFQ-C* Big Five questionnaire for children, *YAM-5* youth anxiety measure for DSM-5.

†*p* < .05, *p < .01, **p < .001

^‡^Cronbach’s alphas could not be calculated, because these measures only consist of one item

Means not sharing similar subscripts differ at *p* < .05.

Correlations (corrected for gender and age) among the two measures of self-conscious emotions can be found in Table 2. As can be seen, significant correlations between shame and guilt were found, and this appeared true for the BSGQ-C (*r* = .51) as well as for the YSR (*r* = .38). Further, BSGQ-C shame was stronger correlated with YSR shame (*r* = .63) than with YSR guilt (*r* = .48), although this difference just failed to reach the conventional level of significance (*Z* = 1.95, *p* = .05). Nevertheless, these findings can be taken as tentative support for the convergent validity of both shame measures. This appeared to be different for the guilt measures: here the correlations between BSGQ-C guilt, on the one hand, and YSR guilt (*r* = .42) and YSR shame (*r* = .46), on the other hand, were found to be equally strong (*Z* < 1). No significant correlations were noted between YSR Lack of guilt and other indices of self-conscious emotions.

**Table 2:** Correlations (corrected for gender and age) among all measures

	(1)	(2)	(3)	(4)	(5)	(6)	(7)	(8)	(9)	(10)	(11)	(12)
(1) BSGQ-C Shame	–	–	–	–	–	–	–	–	–	–	–	–
(2) BSGQ-C Guilt	0.51**	–	–	–	–	–	–	–	–	–	–	–
(3) YSR Shame	0.63**	0.46**	–	–	–	–	–	–	–	–	–	–
(4) YSR Guilt	0.48**	0.42**	0.38**	–	–	–	–	–	–	–	–	–
(5) YSR Lack of guilt	−0.14	0.00	− 0.02	−0.05	–	–	–	–	–	–	–	–
(6) BFQ-C Extraversion	−0.25*	− 0.18	−0.32**	−0.12	−0.07	–	–	–	–	–	–	–
(7) BFQ-C Agreeableness	0.11	0.29**	−0.01	0.14	−0.01	0.30**	–	–	–	–	–	–
(8) BFQ-C Conscientiousness	0.13	0.25*	0.00	−0.01	−0.14	0.28*	0.46**	–	–	–	–	–
(9) BFQ-C Neuroticism	0.39**	0.15	0.45**	0.35**	0.01	0.07	−0.22^†^	−0.06	–	–	–	–
(10) BFQ-C Openness/intellect	−0.01	0.22†	−0.13	0.08	−0.03	0.33**	0.45**	0.56**	−0.06	–	–	–
(11) YAM-5 Total anxiety	0.65**	0.34**	0.61**	0.34**	−0.10	−0.27*	− 0.04	0.08	0.53**	−0.04	–	–
(12) YAM-5-I Anxiety disorders	0.61**	0.37**	0.62**	0.42**	−0.07	−0.30**	0.02	0.15	0.55**	0.05	0.89**	–
(13) YAM-5-II Phobias	0.48**	0.19^†^	0.41**	0.12	−0.11	− 0.15	− 0.10	−0.05	0.34**	−0.13	0.82**	0.45**

*BSGQ-C* Brief shame and guilt questionnaire for children, *YSR* youth self-report, *BFQ-C* Big Five questionnaire for children, *YAM-5* youth anxiety measure for DSM-5

^†^ *p* < .05, *p < .01, **p < .001

### Self-conscious Emotions and Anxiety Disorders and Phobia Symptoms

Correlations (corrected for gender and age) between self-conscious emotions and anxiety disorder and phobia symptoms as indexed by the YAM-5 are also shown in Table 2. As can be seen, shame and guilt were in general significantly and positively related to anxiety disorder and phobia symptoms (*r*’s between 0.19 and 0.65, all *p*’s < 0.05). The only exception was the correlation between YSR guilt and YAM-5-II Phobias (*r* = .12). YSR lack of guilt did not show any significant correlation with YAM-5 anxiety scores (all *r*’s between −0.07 and −0.11).

Shame was more strongly linked to symptoms of anxiety disorders and phobias than guilt, and this appeared true when self-conscious emotions were assessed with both the BSGQ-C (all *Z*’s ≥ 3.18, *p*’s < 0.01) and the YSR (all *Z*’s ≥ 3.19, *p*’s < 0.01). Further, when controlling for the shared variance between both self-conscious emotions, shame remained significantly associated with anxiety disorder and phobia symptoms (all *r*’s between 0.39 and 0.59, *p*’s < 0.001), whereas for guilt most correlations with such symptoms attenuated to a non-significant level (*r*’s between −0.07 and 0.14, with the only exception being the correlation between YSR guilt and YAM-5 Total anxiety: *r* = .26, *p* < .01). Note also that in most cases both shame and guilt correlated stronger with YAM-5-I Anxiety disorders than with YAM-5-II Phobias (*Z*’s ≥ 2.51, *p* < .01).

Follow-up correlations computed between self-conscious emotions and separate YAM-5 subscales revealed that both shame and guilt were most strongly correlated with social anxiety (*r*’s being respectively 0.65 and 0.40 for the BSGQ-C and 0.54 and 0.31 for the YSR, all *p*’s ≤ 0.001) and generalized anxiety (*r*’s being respectively 0.54 and 0.32 for the BSGQ-C and 0.55 and 0.41 for the YSR, all *p*’s < 0.001), which are both subscales of YAM-5-I Anxiety disorders.

### Self-Conscious Emotions and Personality Traits

Correlations (corrected for gender and age) between shame and Big Five personality traits showed the predicted pattern (Table 2). That is, shame as assessed by means of both the BSGQ-C and the YSR was positively associated with neuroticism (*r*’s being 0.39 and 0.45, respectively, *p*’s < 0.001) and negatively with extraversion (*r*’s being −0.25, *p* < .01 and −0.32, *p* < .001, respectively). As expected, guilt as measured with the BSGQ-C was positively associated with the personality trait of agreeableness (*r* = .29, *p* < .001), but also with conscientiousness (*r* = .25, *p* < .01) and openness/intellect (*r* = .22, *p* < .05). In contrast, guilt as assessed by the YSR was not significantly linked to agreeableness (*r* = .14), but only showed a significant positive correlation with neuroticism (*r* = .35, *p* < .001).

### Unique Contributions of Shame and Guilt to Anxiety Symptoms

To examine unique contributions of self-conscious emotions to anxiety disorder symptoms, hierarchical regression analyses were conducted in which we controlled for gender and age on step 0, Big Five personality traits were entered on step 1, and BSGQ-C shame and guilt or YSR shame, guilt, and lack of guilt were added to the model on step 2. As can be seen in Table 3, the analyses revealed a rather consistent pattern of results. Of the personality traits that were entered, neuroticism was positively related to the YAM-5 total anxiety score (β = 0.55, *p* < .001) as well as to the separate scores of anxiety disorders (β = 0.60, *p* < .001) and phobias (β = 0.33, *p* < .001), whereas extraversion was negatively associated with YAM-5 total anxiety (β = −0.36, *p* < .001) and symptoms of anxiety disorders (β = −0.45, *p* < .001). Other traits were not significantly linked to anxiety; only in the case of anxiety disorder symptoms, agreeableness (β = 0.17, *p* < .05) and conscientiousness (β = 0.21, *p* < .01) made positive contributions. In total, Big Five personality traits accounted for between 12% (phobias) and 45% (anxiety disorders) of the variance in YAM-5 scores.

**Table 3:** Results of the hierarchical regression analysis predicting young adolescents’ anxiety symptoms from Big Five Personality traits and self-conscious emotions as indexed by either the BSGQ-C or by items taken from the YSR

	YAM-5 Total anxiety				YAM-5-I Anxiety disorders				YAM-5-II Phobias			
	B	SE	β	∆R^2^	B	SE	β	∆R^2^	B	SE	β	∆R^2^
Step1	–	–	–	0.35**	–	–	–	0.45**	–	–	–	0.12*
BFQ-C Extraversion	−0.85	0.17	−0.36**	–	−0.65	0.10	−0.45**	–	−0.20	0.11	−0.16	–
BFQ-C Agreeableness	0.32	0.20	0.13	–	0.25	0.12	0.17†	–	0.07	0.13	0.06	–
BFQ-C Conscientiousness	0.40	0.21	0.16	–	0.33	0.12	0.21*	–	0.07	0.13	0.05	–
BFQ-C Neuroticism	1.41	0.18	0.55**	–	0.97	0.10	0.60**	–	0.44	0.11	0.33**	–
BFQ-C Openness/intellect	−0.08	0.19	−0.03	–	0.05	0.11	0.03	–	−0.13	0.12	−0.10	–
Step 2	–	–	–	0.10**	–	–	–	0.05*	–	–	–	0.10**
BSGQ-C Shame	1.95	0.39	0.40**	–	0.90	0.24	0.30**	–	1.05	0.26	0.41**	–
BSGQ-C Guilt	− 0.02	0.42	− 0.00	–	0.03	0.27	0.01	–	− 0.05	0.28	− 0.02	–
Or												
Step 2	–	–	–	0.07*	–	–	–	0.06*	–	–	–	0.05^†^
YSR Shame	5.77	1.49	0.31**	–	3.13	0.89	0.27*	–	2.64	0.99	0.27*	–
YSR Guilt	0.42	1.48	0.02	–	1.29	0.88	0.10	–	−0.88	0.98	−0.08	–
YSR Lack of guilt	−1.85	1.33	−0.09	–	−0.70	0.74	−0.05	–	−1.15	0.88	−0.10	–

*BSGQ-C* Brief shame and guilt questionnaire for children, *YSR* youth self-report, *BFQ-C* Big Five questionnaire for children, *YAM-5* Youth anxiety measure for DSM-5

† *p* < .05, * *p* < .01, ** *p* < .001

In all analyses, we controlled for gender and age on step 0

The self-conscious emotions that were added to the regression model on step 2 accounted for 5–10% of additional variance in anxiety scores. However, as shown in Table 3, only shame, either measured by the BSGQ-C or by the YSR, was found to make a significant contribution: all betas were positive, indicating that higher levels of shame were associated with higher levels of anxiety and this was true for the YAM-5 total anxiety score (β’s of 0.40 and 0.31, *p*’s < 0.001) as well as the separate scores of anxiety disorders (β’s of 0.30 and 0.27, *p*’s < 0.01) and phobias (β’s of 0.41 and 0.27, *p*’s < 0.01).

## Discussion

The present study examined the relationships between the self-conscious emotions of shame and guilt, Big Five personality traits, and anxiety disorder symptoms in a sample of non-clinical adolescents. The results first of all clearly indicated that shame (either assessed with the BSGQ-C or the YSR) was positively associated with a broad range of anxiety symptoms, with the strongest correlations being found for symptoms of social anxiety and generalized anxiety disorder. These findings are well in line with Paulus et al. [31] as well as with other studies conducted in adult [9–19] and youth populations [29, 32], and thus provide further support for the notion that shame is not only relevant for psychopathological conditions such as depression [5] and externalizing problems such as anger and aggression [6], but also appears to play a prominent role in anxiety pathology. Further, as anticipated, the evidence for the link between guilt and anxiety symptoms was not convincing. Although correlations between guilt and anxiety symptoms were positive and significant, most of them attenuated to a non-significant level once controlling for the influence of shame. This is consistent with the idea that guilt—at least in non-clinical samples—is in essence benign in nature, and that this emotion only becomes maladaptive when being excessive, ruminative, and fused with shame [2, 8].

The pattern of correlations between self-conscious emotions and Big Five personality traits was also largely as hypothesized. That is, in line with Paulus et al. [31] shame (either assessed with the BSGQ-C or the YSR) was positively correlated with neuroticism, but at the same time also appeared negatively related to extraversion [14, 37–39]. This finding was anticipated because shame is associated with negative affect (which is also a key feature of neuroticism) and docility and submissiveness (which are antagonists of extraversion). Correlations between guilt and Big Five traits were dependent on the measure used to assess this self-conscious emotion. When employing the BSGQ-C the expected positive link with agreeableness was found [37, 38, 40] as well as positive relations with conscientiousness and openness/intellect). This fits with the idea that guilt is associated with prosocial behavior and empathy thereby facilitating interpersonal contact [55, 56], but also seems to indicate that this self-conscious emotion more often occurs in individuals with an in general more positive personality profile. However, when assessed with the YSR, guilt appeared to be only positively correlated with neuroticism, pointing out a link with a less favorable personality. The latter finding has probably to do with the validity of the YSR as a measure of guilt, which is an issue to which we will return later.

When looking at Big Five personality traits as correlates of anxiety disorders symptoms, it became clear that in particular neuroticism was relevant. That is, high levels of neuroticism were associated with higher symptom levels of major anxiety disorders as well as phobias. Further, in the case of major anxiety disorders, the contribution of extraversion was also significant: lower levels of this personality trait were accompanied by higher levels of symptoms. The “vulnerable” personality constellation of high neuroticism and low extraversion has also emerged in a large meta-analytic study linking personality traits to anxiety disorders as well as a number of other internalizing psychopathologies (e.g., mood disorders, obsessive–compulsive disorder [33]).

An important finding of the present study was that shame, even after controlling for the Big Five personality traits, still accounted for a significant proportion of the variance in anxiety disorders symptoms. This result is well in line with that of Paulus et al. [31] and suggests that although shame to some extent overlaps with neuroticism and (low) extraversion, this self-conscious emotion still has exclusive features that are uniquely linked to anxiety pathology. Several scholars have put forward that the etiology of anxiety disorders and many other forms of psychopathology can best be represented as a hierarchical model [26, 57]. The “Big” personality traits such as neuroticism and (low) extraversion can be regarded as general risk factors that predispose individuals to a broad range of disorders, but at a lower level more specific risk factors operate representing the characteristic pathogenic processes for a particular group of disorders. Using their cross-sectional data obtained in 97 inpatient adolescents, Paulus et al. [31] found support for a model in which shame acted as such a lower-order risk factor connecting the general personality trait of neuroticism and anxiety symptoms. However, it is clear that future studies employing prospective research designs are needed to further test the validity of such a mediational scenario.

The current study also provided support for the BSGQ-C being a reliable and valid index of self-conscious emotions in youths. In agreement with Novin and Rieffe [42] who were the developers of this concise self-report measure, findings signified satisfactory reliability (internal consistency) for both the shame and guilt subscales and good validity as was evident from the pattern of correlations with personality traits and the anxiety questionnaire: shame emerged as the more “bad and ugly” self-conscious emotion that was associated with an unfavorable constellation of personality traits and high anxiety levels, whereas guilt appeared to be the “good” emotion that was associated with more advantageous personality traits and no obvious link with anxiety [2, 4].

There were clear indications that the assessment of self-conscious emotions with items of the YSR (as was done in our previous study [32]) was less optimal. The shame item of the YSR and the shame subscale of the BSGQ-C were substantially correlated (*r* = .63) and also yielded comparable results in terms of relations with personality traits and anxiety. Therefore, it seems reasonable to assume that the validity of this YSR item is fiduciary. In contrast, the correlation between the guilt item of the YSR and the guilt subscale of the BSGQ-C was more modest (*r* = .42), but more importantly their pattern of relations with personality traits was quite different: YSR guilt correlated positively with neuroticism, whereas BSGQ-C guilt correlated positively with agreeableness, conscientiousness and openness/intellect. This demonstrates that both indices of guilt seem to measure quite different aspects of this self-conscious emotion: it is most likely that the BSGQ-C is more capable of measuring the reparative action and the empathy associated with guilt, whereas the YSR item mainly taps excessive or pathological guilt feelings and thus may be more fused with shame (see [58] for a good discussion of the difference between affect and action tendencies associated with self-conscious emotions). For the link with anxiety symptoms, this distinction did not really matter: that is, once controlling for shame, both BSGQ-C guilt and YSR guilt were no longer significantly associated with YAM-5 scores.

There might be another relevant distinction between the BSGQ-C and the YSR items. That is, the BSGQ-C [42] is derived from and similar to the Test of Self-Conscious Affect for Children/Adolescents [59–61], which is considered to be an index of trait-like self-conscious emotions, namely shame- and guilt-proneness. The shame, guilt, and lack-of-guilt items of the YSR refer to a fixed time frame of (the past) 6 months, and as such can best be interpreted as state-like indices of these self-conscious emotions. This notion could be investigated in future studies by examining the stability of BSGQ-C and the YSR items over longer time periods.

An additional finding of the present investigation concerned the gender differences that were observed for self-conscious emotions as well as a number of other variables. More precisely, in consonance with what has been reported previously, girls reported somewhat higher levels of shame and guilt (as assessed with the BSGQ-C) [41], agreeableness [62], and anxiety disorders symptoms [63] as compared to boys. Further, boys rated themselves as higher on extraversion than did girls, which is a finding that has received some support in the literature [64]. However, there are also indications that the reverse might be true (i.e., girls scoring higher on extraversion than boys [62]). This divergence has probably to do with different ways by which extraversion has been operationalized: sometimes extraversion is mainly defined as sociability (leading to higher scores in girls), while on other occasions this trait is more described in terms of assertiveness and energy (leading to higher scores in boys).

It should be acknowledged that the current study suffers from a number of limitations. First of all, it should be borne in mind that this investigation was cross-sectional in nature, implying that no causal inferences can be drawn from these data. As noted earlier, longitudinal studies are needed to examine the precise contribution of self-conscious emotions, in particular shame, to anxiety symptoms in youths. A second drawback is concerned with the fact that we only employed self-report questionnaires to measure the relevant constructs. Although this method is pre-eminently suitable for assessing covert phenomena such as self-conscious emotions and anxiety, the inclusion of parent-report could have provided important cross-validational information. A third and final shortcoming pertains to characteristics of the present sample. This was a fairly small, non-clinical sample with a predominantly Caucasian background, and nearly half of the youths who were approached did not participate in the survey. It would be worthwhile to replicate the study in a more representative population of adolescents with a more diverse background and in young people who are referred to a clinical facility.

## Summary

In spite of these limitations, the present study provides further support for the idea that (high) shame plays a significant role in anxiety pathology. Just like Paulus et al. [31], the results indicated that this self-conscious emotion accounted for a unique proportion in the variance of anxiety disorders symptoms, even after controlling for the personality trait of neuroticism, which is known to be a powerful predictor of this type of internalizing problems [33]. Further work is required to clarify the precise role of shame in the etiology of anxiety disorders. In the meantime, we can speculate on how this knowledge can be practically exploited. Two possibilities suggest themselves. To begin with, one could increase awareness of shame in parents, other caregivers, and teachers, as this might be helpful with the identification of youngsters with anxiety problems. Another way to go has to do with the prevention and early intervention of anxiety disorders in young people. Available programs mainly focus on the primary emotion of fear/anxiety [65], but it is good to know that in some youths the secondary emotion of shame is also at work and might be an appropriate target for intervention. There are indications from clinical studies with adults that feelings of shame can be successfully abolished by cognitive-behavioral therapy [10, 66], and so it seems worthwhile to investigate whether such a specific focus on shame can further improve of our existing prevention and early intervention programs for youths with anxiety problems.
